# Supplementary material for: Novel enzymes for biodegradation of polycyclic aromatic hydrocarbons identified by metagenomics and functional analysis in short-term soil microcosm experiments
Source: Sci Rep. 2024 May 21;14:11608. doi: 10.1038/s41598-024-61566-6 (PMC11109138; doi:10.1038/s41598-024-61566-6)

**Supplementary material**

**Novel enzymes for biodegradation of polycyclic aromatic hydrocarbons identified by metagenomics and functional analysis in short-term soil microcosm experiments**

Kinga K. Nagy^1,2,a^, Kristóf Takács^3,a^, Imre Németh^1,a^, Bálint Varga^3^, Vince Grolmusz^3,4^, Mónika Molnár^1,*^, Beáta G. Vértessy^1,2,*^

a: joint first authors , * : corresponding authors (molnar.monika@vbk.bme.hu, vertessy.beata@ttk.hu)

Affiliations

1: Department of Applied Biotechnology and Food Science, Faculty of Chemical Technology and Biotechnology, Budapest University of Technology and Economics, Műegyetem rkp. 3., H-1111 Budapest, Hungary

2: Institute of Enzymology, Research Centre for Natural Sciences, Magyar tudósok körútja 2., H-1117, Budapest, Hungary

3: PIT Bioinformatics Group, Eötvös Loránd University, H-1117 Budapest

4: Uratim Ltd, H-1118 Budapest

Table of contents

[Table S1: Ring cleavage dioxygenases 2](#_Toc136946483)

[Table S2: Bacterial peroxidases 3](#_Toc136946484)

[Table S3: Other known bacterial PAH degrading enzymes 3](#_Toc136946485)

[Table S4: Bacterial ring-hydroxylating oxygenases 3](#_Toc136946486)

[Table S5 Bacterial fluorene metabolism 4](#_Toc136946487)

[Table S6 Bacterial phenanthrene degrading enzymes 4](#_Toc136946488)

[Table S7 Amino acid sequences of the hits 5](#_Toc136946489)

[Figure S1 Disorder predictions of the selected proteins.. 6](#_Toc136946490)

# PAH-degrading enzyme families used for HMM profile preparation

## Table S1: Ring cleavage dioxygenases

| ID |  | Accession | Alternate Acc. | Function | Species |
| --- | --- | --- | --- | --- | --- |
| 1p1 |  | B0BK98 | B0BK98_9MICC | Cupin domain-containing protein | Pseudarthrobacter phenanthrenivorans |
| 1p2 |  | B0BK99 | B0BK99_9MICC | Dioxygenase | Pseudarthrobacter phenanthrenivorans |
| 1p3 |  | O24721 | PHDI_NOCSK | Dioxygenase | Nocardioides sp. |
| 1p4 |  | C0KUL5 | C0KUL5_9MYCO | 1-hydroxy-2-naphthoic acid dioxygenase | Mycobacterium sp. CH1 |
| 1p5 |  | Q51493 | NDOA_PSEAI | Naphthalene 1,2-dioxygenase system | Pseudomonas aeruginosa |
| 1p6 |  | Q67FT0 | Q67FT0_PSESE | Gentisate 1,2-dioxygenase | Pseudaminobacter salicylatoxidans |
| 1p7 |  | Q75W71 | Q75W71_9RHIZ | Gentisate 1,2-dioxygenase | Xanthobacter polyaromaticivorans |
| 1p8 |  | D3QJT0 | D3QJT0_ECOCB | Gentisate 1,2-dioxygenase | Escherichia coli |
| 1p9 |  | B1K9G3 | B1K9G3_BURCC | Gentisate 1,2-dioxygenase | Burkholderia cenocepacia |
| 1p10 |  | YP_004232675.1 | WP_013592695 | Gentisate 1,2-dioxygenase | Acidovorax_avenae |

## Table S2: Bacterial peroxidases

| ID | Accession | Alternate Acc. | Function | Species |  |
| --- | --- | --- | --- | --- | --- |
| 2p1 | Q9R2E9 | KATG_MYCVN | Catalase-peroxidase | Mycobacterium vanbaalenii |  |
| 2p2 | S5TTY5 | S5TTY5_9GAMM | Catalase-peroxidase | Cycloclasticus zancles |  |
| 2p3 | A0A2A9KHU3 | A0A2A9KHU3_9BURK | Catalase | Collimonas sp. |  |

## Table S3: Other known bacterial PAH degrading enzymes

| ID | Accession | Alternate Acc. | Function | Species |
| --- | --- | --- | --- | --- |
| 3p1 | P0A110 | NDOB_PSEPU | Naphthalene 1,2-dioxygenase system | Pseudomonas putida |
| 3p2 | Q9WXG7 | DPDD_ALCFA | Cis-3,4-dihydrophenanthrene-3,4-diol dehydrogenase | Alcaligenes faecalis |
| 3p3 | Q79EM8 | PHDJ_NOCSK | Trans-2'-carboxybenzalpyruvate  hydratase-aldolase | Nocardioides sp. |
| 3p4 | Q52126 | NDOR_PSEPU | Naphthalene 1,2-dioxygenase system | Pseudomonas putida |
| 3p5 | P74308 | AKR_SYNY3 | Aldo/keto reductase | Synechocystis sp. |

## Table S4: Bacterial ring-hydroxylating oxygenases

| ID | Accession | Alternate Acc. | Function | Species |
| --- | --- | --- | --- | --- |
| 4p1 | O87616 | O87616_PSEAI | Putative regulatory protein  for oxygenase | Pseudomonas aeruginosa |
| 4p2 | A0A0Q8E065 | A0A0Q8E065_9ACTN | Ring-hydroxylating oxygenase | Nocardioides sp. |
| 4p3 | B3RBC6 | B3RBC6_CUPTR | Subunit of multicomponent oxygenase | Cupriavidus taiwanensis |
| 4p4 | G9G360 | G9G360_9BACT | Ring-hydroxylating dioxygenase | uncultured bacterium |
| 4p5 | G9G301 | G9G301_9BACT | dioxygenase alpha subunit | uncultured bacterium |
| 4p6 | H2IMD2 | H2IMD2_VIBSJ | ring-hydroxylating dioxygenase | Vibrio sp. |
| 4p7 | A1K8H8 | A1K8H8_AZOSB | Putative ring hydroxylating | Azoarcus sp. |

## Table S5 Bacterial fluorene metabolism

| ID | Accession | Alternate Acc. | Function | Species |
| --- | --- | --- | --- | --- |
| 5p1 | Q93UV4 | FLNB_TERSD | Fluoren-9-ol dehydrogenase | Terrabacter sp |
| 5p2 | P77567 | NHOA_ECOLI | N-hydroxyarylamine O-acetyltransferase | Escherichia coli |

## Table S6 Bacterial phenanthrene degrading enzymes

| ID | Accession | Alternate Acc. | Function | Species |
| --- | --- | --- | --- | --- |
| 6p1 | G4WYQ4 | G4WYQ4_9SPHN | Phenanthrene dioxygenase | Novosphingobium sp. |
| 6p2 | M4VW12 | M4VW12_9BURK | Phenanthrene dioxygenase | Burkholderia sp. |
| 6p3 | Q9WXG7 | DPDD_ALCFA | Cis-3,4-dihydrophenanthrene-3,4-diol  dehydrogenase | Alcaligenes faecalis |
| 6p4 | Q79EM8 | PHDJ_NOCSK | Trans-2'-carboxybenzalpyruvate  hydratase-aldolase | Nocardioides sp. |
| 6p5 | Q79EM7 | PHDK_NOCSK | 2-formylbenzoate dehydrogenase | Nocardioides sp. |
| 6p6 | P0A110 | NDOB_PSEPU | Naphthalene 1,2-dioxygenase system | Pseudomonas putida |
| 6p7 | Q8G8B6 | CARAA_PSERE | Carbazole 1,9a-dioxygenase | Pseudomonas resinovorans |

## Table S7 Amino acid sequences of the hits

| **Identifier** | **Protein sequences** | **Lenght  (AS)** | **BLAST first hit** | **% match with BLAST's first hit** |  |
| --- | --- | --- | --- | --- | --- |
|  |  |  |  |  |  |
| PAH1_16 | MEAVTKTPEREAFYKKIDGENLTALWTVMSDLITPEPKSACRPHLWKFDVIRDYMREAGKLITAKEAERRVLVLENPGLRGQSRITTSLYAGVQMVVPGDVAPAHRHSQSALRFVLEGKGAHTAVDGERTAMEPGDFIITPSMTWHDHSNETDQPMFWLDGLDIPLVQFFDCSFAEGSKEDQQTITKPAGDSFARYGHNLLPVDVKRSSKTSPIFSYPYAHTREALEKARASEEWDACHGLKLKFSNPETGDFAMPTIGTFIQLLPKGFKTARYRSTDATVFCPIEGHGRSRIGDAVFEWGPRDLFVVPSWQWVTHEAEDDAVLFSFSDRPVQQKLDLFREDRGNA | 346 | gentisate 1,2-dioxygenase [Bradyrhizobium sp. S23321] | 98.00% |  |
| PAH1_17 | MEAVQKTPEREAFYKKIDGENLSALWNVMGDLITPEPKSACRPHLWKFDAIRDYMTEAGKLITAKEAERRGLVLENPGLRGQSKITTSLFAGVQMVVPGDIAPAHRHSQSALRFVLEGKGAYTAVDGERTAMEPGDFVITPSMTWHDHSNETSEPMFWLDGLDIPMVQFFDASFAEGSNEDQQKITRPAGDSFARYGHNLLPVDEKRTSKTSPIFNYPYSYTREALEQAKTRNEWDACHGLKLKFSNPETGDFAMPTIGTFIQLLPKGFKTARYRSTDATVFAAIEGRGRTRIGEQTFEWGPRDLFVVPSWQWVTHEADADSVLFSFSDRPVQQKLDLFREDRGNA | 346 | gentisate 1,2-dioxygenase [Bradyrhizobium lablabi] | 99.00% |  |
| PAH1_99 | MKNDLIPSPVRLHAVAGHGQPDPTPELEQLYRGFEEELLVPLWTEIGDLMPRQPKSKAVPHVWRWERLKALAAQAGEIVPVGRGGERRAIALANPALGGRPFATPTLWAAIQYLMPGEDAPEHRHTQHAFRFVVEGDGVWTVVNGDAVRMSRGDFLPQAGWNWHAHHNAATAPMAWIDGLDIPFSYYSESQFFEVGRDKISQAERTTAERSYSERLWAHPGLRPVSSTAATAATPLLAYRWVDTDRALADQLALEDEGQAGTLSHGHAAVRFTNPTTGGDVLPTMRCEMHRIRAGGKTKTTREVGSSVYQVFDGEGIVTVGDRTWQVTRGDLFVVPSWASFAVNALEASNLDLFRFGDAPIFDALHNYRTEIIS | 374 | cupin domain-containing protein [Acidovorax sp. OV235] | 86.00% |  |
| PAH1_102 | MSNHDGFQQAPVHNAMAPDDSPELRQLYADFEAGHMMPLWTQIGNLMPKHPMPRAVPHVWKWSDLYPLAKRSGDLVPVGRGGERRAIGLGNPGLEGRPYISPTLWCAIQYLGPRETAPEHRHAQNAFRFVIEGEGVWTVVNGDPVRMSRGDLLLTPGWNFHGHQNVTDKPMAWIDGLDIPFSYQNDVGFFEFGSENLTDITTPQYSRGERLWCHPGLRPLSGLANTVSSPIGAYRWEHTDRALDEQLRLEEEGFPGVQEKGHAAVRFINPTTGGDIMSSIRAEFHRLRAGAVTAERREVGSRVFQVFEGRGQVMLDGVTRHLEKGDLFVVPSWISWSLQAESQFDLFSFSDAPIMADVDENRLIVAHTLKSWQVAGQSRASAYGLAAPERPIRPTGH | 397 | cupin domain-containing protein [Pseudomonas sp. GM55] | 96.00% |  |
| PAH1_105 | MNTATLRAAPPQADERRAYYERIRPLNLTPLWESLHALVPREPQTPCVPALWRYDDIRPLLMESAELITADEAVRRVLVLENPAIPGRSSITQSLYAGLQLIMPGEVAPSHRHVQSALRFIVDGKGAYTTVGGERTTMHPGDFIITPSWAWHDHGNEGIEGVSEPVVWLDGLDIPMVRFFDAGFAENAEAKVQHVARPEGHSLARFGHNMVPVRHDHTSATSPIFNYPYLRSREALAQLQMQEAPDAWLGHKLRYINPLTGGSPMPTIATNLQLLPRGFAGKTHRMTDGAVYSVVEGRGHADIGGQRFDFGPRDTFVVPSWAPLKLVASDDVVLFSFSDRPVQQAMGVLREAFLED | 356 | gentisate 1,2-dioxygenase [Variovorax sp. YR216] | 91.00% |  |
| PAH1_117 | MPVIPNPKAVPHVWKWSRLYPLAERSGDLVPVGRGGERRAIGLSNPGLGGRAYVSPTLWAAIQYLGPRETAPEHRHAQNAFRFVVEGEGVWTVVNGDPVRMSRGDLLLTPGWNFHGHHNDTDHPMAWIDGLDIPFSYQNDVGFFEFGSDRVTDYATPQFSRGERLWAHPGLRPLSQLTDTVSSPLAAYRWEFTDRALTEQLLLEDEGQPATVGQGHAAIRYVNPTTGGDVMPTIRCEFHRLREGTVTPPRREVGSSVFQVFEGTGSVVLNGTETKLEKGDMFVVPSWVACSLQAETRFDLFRFSDAPIIERLGFARTLVENNER | 324 | cupin domain-containing protein [Mesorhizobium australicum] | 89.00% |  |
| PAH6_39 | MDNQPSTAGKCPFMHGGNTSAAASNMDWWPNALNLDILHQHDTKTNPLGADFNYAEEFKKLDLDAVKKDLHAFMTDSQEWWPADWGHYGGLMIRMAWHAAGTYRIADGRGGAGTGNQRFAPLNSWPDNVNLDKARRLLWPIKKKYGNKLSWSDLIVLAGTMAYESMGLKVYGFAGGRADIWHPEKDIYWGSEKEWLGNSSRYDGEQRESLENPLAAVQMGLIYVNPEGVNGQPDPLRTAQDIRLTFARMAMNDEETVALTAGGHTVGKCHGNGKAELLGPNPEAADVSEQGFGWHNSNGKGFGRDTVTSGLEGAWTAHPTQWDNGYFYNLFNYEWELKKSPAGAWQWEPINMKEEDKPVDVEDPSIRHNPIMTDADMAMVKDPEYRKISERFYKNQAYFSEVFARAWFKLTHRDLGPKARYLGPDVPQEDLIWQDPVPKVDYTLSDAEIAALKAKLLNSGLSISELVTTAWDSARTFRGSDYRGGANGARIRLTPQKDWQGNEPARLQKVLATLEAIQAGLSQKVSMADLIVLGGTAVVEKAAHDAGVNITVPFAAGRGDATDAMTDAESFAVLEPIHDGYRNWLKNDYAVSAEELLLDRTQLMGLTAHEMTVLVGGMRVLGTNYGGTKHGVLTNREGILTNDFFVNLTDMGNTWKPAGNNLYEIRDRNTGAVKWTATRVDLVFGSNSILRSYAEVYAQDDAKEKFVKDFVQAWTKVMNADRFDLN | 726 | catalase/peroxidase HPI [Methylotenera sp. 24-45-7] | 87.00% |  |
| PAH6_78 | MTEESKCPFHAAGTSGSTTSRDWWPNQLRVDLLNQHSERSNPLGEKFNYAAEFKKLDYSALKADLKALLTDSQDWWPADWGTYTGLFIRMAWHGSGTYRTVDGRGGAGRGQQRFAPLNSWPDNVSLDKARRLLWPVKQKYGQKISWADLMILAGNVALENAGFRTFGFGAGREDVWEPDQDVNWGDEKAWLAHRNPETLAKNPLAATEMGLIYVNPEGPNASGDPLSAAAAIRATFGNMAMDDEEIVALIAGGHTLGKTHGAASASHVGAAPEAAPIEQMGLGWSSSHGSGSGADAITSGLEVVWTQTPTQWSNNFFENLFKFEWVQTRSPAGAIQFEAKDAPEIVPDPFDPAKKRKPTMLVTDLTLRFDPAFEKISRRFLNDPQAFNEAFARAWFKLTHRDMGPKARYIGPEVPKEDLIWQDPLPTPQHQPTTADIADLKAKIAASGLSVSELVSVAWASASTFRGGDKRGGANGARLALAPQKDWAVNAIAVGVLPQLQAIQQASGKASLADVIVLAGVVGVEQAAKAAGVSVQVPFAPGRVDARQDQTDVASFDVMEPVADGFRNYRRVASSTATEELLIDKAQQLTLTAPQLTALIGGLRVLGANYDGSQHGVLTDKVGVLSNDFFVNLLDMGTAWKSVDDTAQVFEGRDRKSGAVKYTATRNDLVFGSNAVLRALAEVYASADAHEKFVRDFVAAWTKVMNLDRFDLAEAPANV | 719 | MULTISPECIES: catalase/peroxidase HPI [Acidovorax] | 99.00% |  |
| PAH6_112 | MENSKATNGGGKCPFVHGANTEVSNAVMDWWPKALNLDILHQHDTKTNPLGADFNYAEEFKKLDLAAVKKDLTALMTDSQDWWPADWGHYGGLMIRMAWHVAGTYRISDGRGGSNTGNQRFAPLNSWPDNVNLDKSRRLLWPIKKKYGNKLSWADLFILAGNMAYESMGLKTFGFAGGRQDIWHPEKDIYWGSEKEWLAETKNRYDNDENRDTLENPLAAVQMGLIYVNPEGVDGVPNPLRTAQDVRTTFKRMAMNDEETVALTAGGHTVGKCHGNGDATILGQSPEGANLEDQGFGWMNPKGKGNAEDTVSSGLEGSWTTNPTRWDNEYFNLLLKYDWELKKSPAGAWQYEPINIAEEDKPFDAHIPNVRRNPIMTDADMALKMDPEYRKISERFHNDQEYFTEVFARAWFKLTHRDLGPKTRYQGPDAPQEDLIWQDPIPAVDYTLSESEIDDLKQTLLNSGLSKTELINTAWDSARTFRGSDYRGGANGARIRLAPQKDWAGNEPERLQKVLNKLTEIQSGWHKKVSIADLIVLGGSAAIEKAAQEAGVNIKVPFSAGRGDATAEMTDVDSFDVLEPLHDAYRNWVKKEYEVNPEELMLDRTQLMGLTAPEMTVLIGGMRVLGANYGGSKHGVFTQKEGVLSNDFFVNLTDMNNSWKPVANNLYNIVDRKTGETKWTATRVDLVFGSNSILRAYAEVYAQDDNKEKFVHDFVAAWNKVMNLDRFDLA | 730 | peroxidase, partial [Lutibacter sp. BRH_c52] | 94.00% |  |

## **Figure S1** Disorder predictions of the selected proteins. **A** predictions for dioxygenases **B** predictions for catalase-peroxidases. The plot shows the IUPRED2 sequence−prediction profile indicated in red, as well as the ANCHOR2 prediction shown in blue. Residues with scores above 0.5 are predicted as disordered.


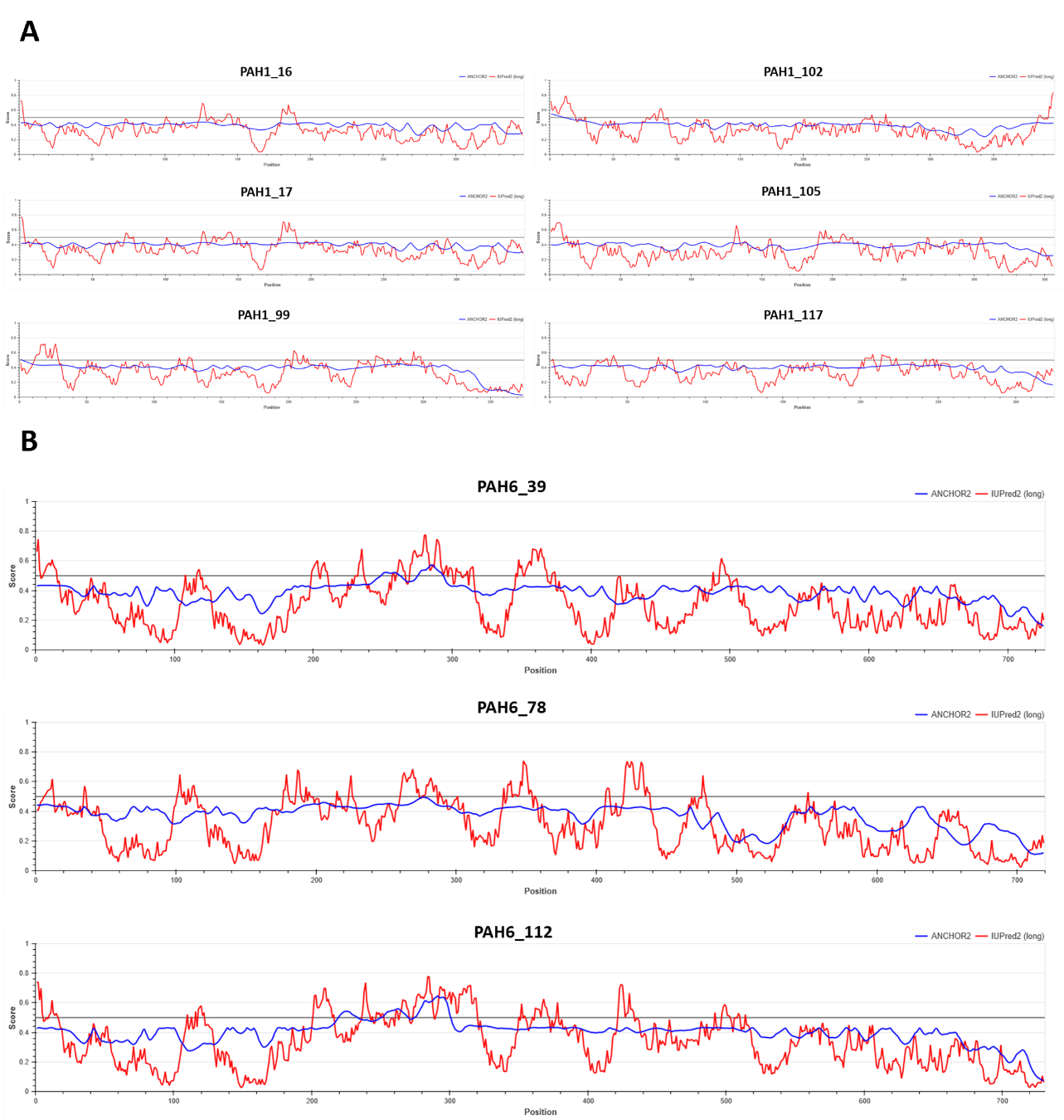

Supplement: Supplementary file 1 — Supplementary Information. [file 41598_2024_61566_MOESM1_ESM.docx]
